# Problems undermining the health-related quality of life of people living with HIV in Spain: a qualitative study to inform the development of a novel clinic screening tool

**DOI:** 10.1186/s12955-022-01978-y

**Published:** 2022-05-25

**Authors:** Kelly Safreed-Harmon, Maria J. Fuster-RuizdeApodaca, Marta Pastor de la Cal, Jeffrey V. Lazarus

**Affiliations:** 1 Barcelona Institute for Global Health (ISGlobal), Hospital Clínic, University of Barcelona, Barcelona, Spain; 2 Faculty of Medicine, University of Barcelona, Barcelona, Spain; 3 Sociedad Española Interdisciplinaria del Sida (SEISIDA), Madrid, Spain; 4 Universidad Nacional de Educación a Distancia (UNED), Madrid, Spain; 5 Bizkaisida, Bilbao, Spain

**Keywords:** Health-related quality of life, HIV, Symptoms, Patient-reported outcome measures, Qualitative research, Spain

## Abstract

**Background:**

In settings with high antiretroviral therapy coverage, numerous health-related issues continue to undermine the health and health-related quality of life (HRQoL) of people living with HIV (PLHIV). As part of a larger study to develop and validate a new patient-reported outcome measure for use in HIV clinical care in Spain, we sought to identify the most burdensome health-related issues experienced by PLHIV in order to determine which issues should be addressed in the new instrument.

**Methods:**

We conducted a literature review and a qualitative study based on four focus group discussions (FGDs) with key informants in Spain. Participants were selected via purposive sampling. Two FGDs convened 16 expert HIV service providers, and two convened 15 PLHIV with diverse epidemiological profiles. FGDs followed semi-structured interview scripts and incorporated an exercise to prioritise the most critical health-related issues among those named in the discussions. Content analysis was conducted using MAXQDA 12.

**Results:**

The analysis of FGD data identified several broad categories of issues that were perceived to negatively affect PLHIV. The most frequently named issues fell within the categories of social problems; physical symptoms; psychological problems; and sexuality-related problems. Regarding social problems, stigma/discrimination was by far the issue raised the most frequently. In the prioritisation exercise, stigma/discrimination was also ranked as the most burdensome issue by both service providers and PLHIV. Within the physical symptoms category, the issues named most frequently were sleep-related problems, fatigue, physical pain and body fat changes. Regarding psychological problems, FGD participants most commonly spoke of emotional distress in general terms, and also called attention to depression and anxiety. In the prioritisation exercise, both service providers and PLHIV ranked psychological well-being as the second-most important issue following stigma. Sexuality-related problems that were reported included sexually transmitted infections, hormonal problems, lack of libido, and general sexual dissatisfaction.

**Conclusions:**

PLHIV are negatively affected by a wide range of health-related issues. HIV-related stigma and psychological well-being remain major challenges. Identifying and addressing these and other issues in routine clinical care supports healthy aging and may ultimately contribute to better health and HRQoL outcomes in this population.

## Introduction

Following the introduction of the first highly effective antiretroviral therapy (ART) regimens in 1996, the widespread use of ART has transformed HIV care [1, 2]. ART suppresses viral replication and thus prevents HIV from destroying the immune system. More than two-thirds of the world’s 38 million PLHIV are now taking ART, and its effect on survival is reflected in the 47% decline in HIV-related deaths from 2010 to 2020 [3]. For people who initiate ART sufficiently early in the course of infection, life expectancy is almost that of the general population [4].

Health systems are increasingly focusing on the question of how to meet the health needs of large numbers of PLHIV who are stable on ART [5]. Viral suppression has conventionally been regarded as a key marker of the success of HIV care, but as more people live with controlled HIV for many years, there is recognition that health-related quality of life (HRQoL) warrants greater consideration [6]. This concern is consistent with the World Health Organization’s definition of health as “a state of complete physical, mental and social well-being and not merely the absence of disease or infirmity” [7]. While a consensus definition of HRQoL is lacking, it has been described in broad terms as “a multidimensional construct concerned with the impact of health on an individual’s perception of their well-being and level of functioning in important areas of their life” [8].

A large British study found that virally suppressed PLHIV reported significantly worse HRQoL than a representative sample of the general population, and other studies have documented similar gaps [9–11]. These outcomes may reflect the challenges of living with HIV on a long-term basis. Although high adherence to ART typically controls HIV infection, PLHIV often experience bothersome physical symptoms such as pain, fatigue and gastrointestinal distress [12–15]. Furthermore, PLHIV have a greater multimorbidity burden than the general population, with comorbidities occurring at younger ages [2]. They are at elevated risk of some comorbidities including osteoporosis, diabetes, cardiovascular disease, and chronic kidney disease [2, 16–18]. HIV remains highly stigmatised, and discrimination and other manifestations of stigma contribute to the social isolation and mental health problems that are commonly reported by PLHIV [19–22]. Periodic disability as well as stigma and discrimination have limited the employment opportunities of many PLHIV, with consequences for their material well-being and long-term financial security [23, 24].

Correlations have been observed between many of these issues and negative HRQoL outcomes in PLHIV populations [25–27]. Also, poorer HRQoL has been observed to predict both hospitalisation and mortality in PLHIV, suggesting that efforts to influence modifiable determinants of HRQoL may have important health benefits [28–30]. Other research in PLHIV has found healthcare providers to have low awareness of patients’ symptoms and of their self-reported drug use [31, 32]. Furthermore, healthcare providers might not be attuned to some health-related issues that can affect the HRQoL of PLHIV. In a United States study in which both PLHIV and HIV care providers were asked to rank their priorities for issues to address in a routine clinic visit, PLHIV expressed greater concern about pain, physical functioning, HIV-related stigma and social support than did providers [33]. A large qualitative study in the United Kingdom and Ireland found that HIV care providers preferred to focus clinical consultations on HIV treatment and physical outcomes while giving less attention to patients’ other concerns, including psychological and social concerns [34]. Taken together, these findings raise the prospect that addressing burdensome health-related issues more effectively as part of routine HIV management might lead to improvements in HRQoL and health outcomes.

Patient-reported outcome measures (PROMs) offer a means of capturing information about a wide range of health-related issues, and administering PROMs in routine clinical care can lead to the recognition of problems that might have otherwise been overlooked [35]. A large number of non-disease-specific PROMs have been designed to serve the general patient population. For example, the PROMIS initiative has published more than 100 validated PROMs that can be used across different areas of healthcare to obtain patient-reported information on issues such as alcohol use, cognitive function, emotional distress, pain, sleep, sexual function, self-efficacy and social isolation [36]. In some contexts, it may be preferable to administer disease-specific PROMs that have been designed to more fully capture experiences associated with particular conditions. Reflecting the diverse ways in which living with HIV can affect people physically, emotionally and socially, a 2017 review identified more than 100 validated HIV-specific PROMs addressing concerns such as symptoms, psychological challenges, HIV-related stigma, body appearance, and social support [37].

Given the many potential health-related challenges facing PLHIV, time and logistical constraints point to the need for short broadly-focused PROMs that can be used to identify multiple types of issues in routine clinical care. Researchers have reported on the development of two such instruments in recent years, with one informed by qualitative research in the United Kingdom and Ireland, and the other informed by qualitative research in Canada [38, 39]. It is not known to what extent these instruments capture the health-related concerns of PLHIV in countries with different social, cultural, epidemiological and health system dynamics.

This study is part of a larger study conducted for the purpose of developing a short broadly focused PROM for use in HIV clinical care in Spain. It is envisioned that the new PROM, the HIV Clinic Screening Tool (CST-HIV), will assist healthcare providers in identifying health-related issues that PLHIV perceive to be burdensome, creating more opportunities to address these issues through interventions and referrals. Future research is planned to determine whether the use of the CST-HIV in HIV clinical care predicts better health and HRQoL outcomes. The development and piloting of the CST-HIV initially was reported in 2021 [40]. The current article complements the earlier article by providing further details about the first two steps of instrument development, and in doing so demonstrates how this formative research contributed to the content validity of the CST-HIV. The overall objective in these two steps was to identify the most burdensome health-related issues experienced by PLHIV in order to determine which issues should be addressed by the new PROM. Specific objectives were to: (a) conduct a literature review that would inform qualitative data collection and subsequent phases of instrument development; and (b) obtain qualitative evidence from PLHIV and HIV service providers.

## Methods

In accordance with established methodologies for instrument development [41], our study employed a four-step process: (1) a literature review; (2) a qualitative study using data from key informants; (3) item pool development; and (4) pilot testing. The first two steps are reported in this article. Data collection and analysis for these two steps took place in January–November 2019. Ethical approval for the study was obtained from the research ethics committee of Hospital Clínic Barcelona in 2019, and all study participants provided written informed consent.

### Literature review

We conducted an exploratory literature review to obtain information about issues that undermine the well-being of PLHIV and to identify themes that would warrant further exploration in focus group discussions (FGDs). We searched PubMed using search strings that addressed two major lines of research: the symptom burden in PLHIV and predictors of HRQoL in PLHIV. The search strings employed both general descriptors and MeSH terms (“Annex 1”). We also searched PubMed for studies relating to HIV symptom screening tools and the use of HIV-specific PROMs in clinical practice. We reviewed the references cited in key articles to identify further relevant sources of information, as well as incorporating other sources known to research team members through prior work. We used Scopus and ResearchGate to identify articles that cited the widely used HIV Symptom Index as a key source and considered the relevance of those articles as well [42].

We restricted the selection of studies relating to the symptom burden in PLHIV to English-language peer-reviewed articles that reported on adult PLHIV who live in high-income countries and are taking ART. We prioritised articles reporting on the symptom burden in PLHIV from 2010 onward, in recognition that the symptom profile has changed in accordance with ART improvements. Articles were excluded if they focused on only one type of symptom such as gastrointestinal distress. Although the search criteria initially called for only peer-reviewed articles to be included in literature review findings, the decision was made to also include conference abstracts due to the paucity of relevant research identified in the peer-reviewed literature. Other inclusion and exclusion criteria applied in the initial search were also applied to conference abstracts. Title/abstract screening and full-text screening were performed in the same stage of the review process. Data from selected articles and abstracts were compiled in a Microsoft Word table.

The HRQoL search in PubMed identified a very large number of records, including a 2014 narrative review article reporting on HRQoL in PLHIV in high-income countries [25]. We thus modified our search to identify articles published from 2013 onward in order to supplement the findings of the review article. Articles were included if they presented original research or systematic review findings about factors associated with HRQoL outcomes in adult PLHIV in high-income countries. Articles were excluded if validated HRQoL instruments had not been used to obtain HRQoL outcomes. Articles were also excluded if the focus of the research was on the relationship between HRQoL and one specific health condition (e.g., pain); if the study population was restricted to individuals with a specific comorbidity in addition to HIV (e.g., hepatitis C virus); if the study population had recently initiated antiretroviral therapy; or if the findings were obtained from a clinical trial. Conference abstracts were excluded, as were articles in languages other than English or Spanish. Records retrieved from this search underwent title/abstract screening, and selected records then underwent full-text screening. Data extraction was performed on the articles identified through this process, with data compiled in a Microsoft Word table.

### Qualitative study

For the qualitative study we conducted focus group discussions (FGDs) to obtain the perspectives of PLHIV and other key informants regarding the most burdensome health-related problems facing PLHIV. The methodologies for data collection and analysis followed standard qualitative research procedures [43].

Two FGDs enrolled HIV service providers (N = 8 per FGD) and two FGDs enrolled PLHIV (N = 8 and N = 7). We selected participants in the service provider FGDs via purposive sampling to ensure the representation of different types of providers such as physicians, nurses, psychiatrists, psychologists and representatives of nongovernmental organisations (NGOs). Service providers worked in the metropolitan areas of Madrid, Barcelona, Bilbao, Sevilla and Valencia. We selected participants in the PLHIV FGDs via purposive sampling to ensure diverse epidemiological profiles in terms of age, sex, sexual orientation, and history of drug use. One PLHIV FGD was comprised of clients of an NGO providing HIV services in Barcelona, and the other PLHIV FGD was comprised of patients at the HIV outpatient clinic of a large Barcelona university hospital.

We conducted the FGDs in April and May 2019, with each one lasting approximately two hours. All FGDs took place in Spanish. Two members of the research team facilitated each FGD. The primary facilitator was always the same person, an expert researcher in HIV and HRQoL. The facilitators previously knew many but not all of the individuals who participated in the HIV service provider FGDs. The facilitators had no previous relationship with any of the individuals who participated in the PLHIV FGDs.

The facilitators used semi-structured scripts with open-ended questions and prompts to guide the discussions, which primarily were centred on identifying and discussing the health-related problems thought to have the greatest negative effect on the quality of life of PLHIV. Questions are summarized in Table 1 and focus group scripts are provided in “Annex 2”. Participants in all four focus groups also were asked to carry out a prioritisation exercise in which they selected what they believed to be the most burdensome issues from among all issues identified by their group. To prepare for this exercise, one facilitator noted specific burdensome issues on a flip-chart upon first mention of each issue. Participants then were asked to select their priority issues from the full list. They indicated their decisions by placing green stickers provided by the facilitators next to the items on the flip-chart.

**Table 1:** Summary of focus group discussion questions

HIV service provider focus groups	People living with HIV focus groups
1. In your opinion, what health-related problems have the greatest negative effect on the quality of life of people with HIV? 2. Among the health-related problems that you have identified, which ones are most important to include in a short clinic screening tool? 3. Are you generally able to identify any of these problems in your patients/clients? If yes, how? 4. What interventions do you provide to respond to these problems?	1. What health-related problems have the greatest negative effect on your quality of life? 2. Of all the problems you have mentioned, what do you think are the most important, considering not only yourself but people with HIV in general? 3. Do you discuss these problems with your doctor or other healthcare provider? If you discuss these problems, how are they raised, and is your healthcare provider able to help? If you do not discuss these problems, why not? 4. What do you think your doctors or other healthcare providers could do to help you solve or more effectively manage the problems you mentioned?

We performed a qualitative analysis through a directed content analysis of the FGD transcripts [44] assisted by the qualitative analysis software MAXQDA 12. For this purpose, FGDs were recorded, transcribed literally, reviewed for accuracy, and coded. Inductive and deductive coding were used to identify relevant concepts, and an analysis of these concepts led to the identification of key categories and subcategories of health-related problems. There was also a quantitative analysis of the qualitative data to determine the number of times each code and category was used. Three research team members who were experts in HIV-related public health and psychosocial issues, two of whom had community-level experience working with PLHIV, assessed the methodological and theoretical quality of core categories and subcategories [45]. For the prioritisation exercise that drew on the flip-chart lists generated during focus group discussions, the research team coded the frequencies for each FGD in a Microsoft Excel document.

Illustrative quotations for key themes were translated into English for reporting purposes.

## Results

### Literature review

The literature review identified seven studies that met inclusion criteria regarding the symptom burden in relevant PLHIV populations [13, 46–51]. Three types of symptoms were reported to be common across many of these studies: sleep-related symptoms, fatigue/energy-related symptoms, and muscle/joint pain. Other symptoms noted to be common in a smaller number of studies included anxiety/fear, sadness/depression, peripheral neuropathy, sexual dysfunction, changes in body appearance, and gastrointestinal symptoms.

**Fig. 1:**
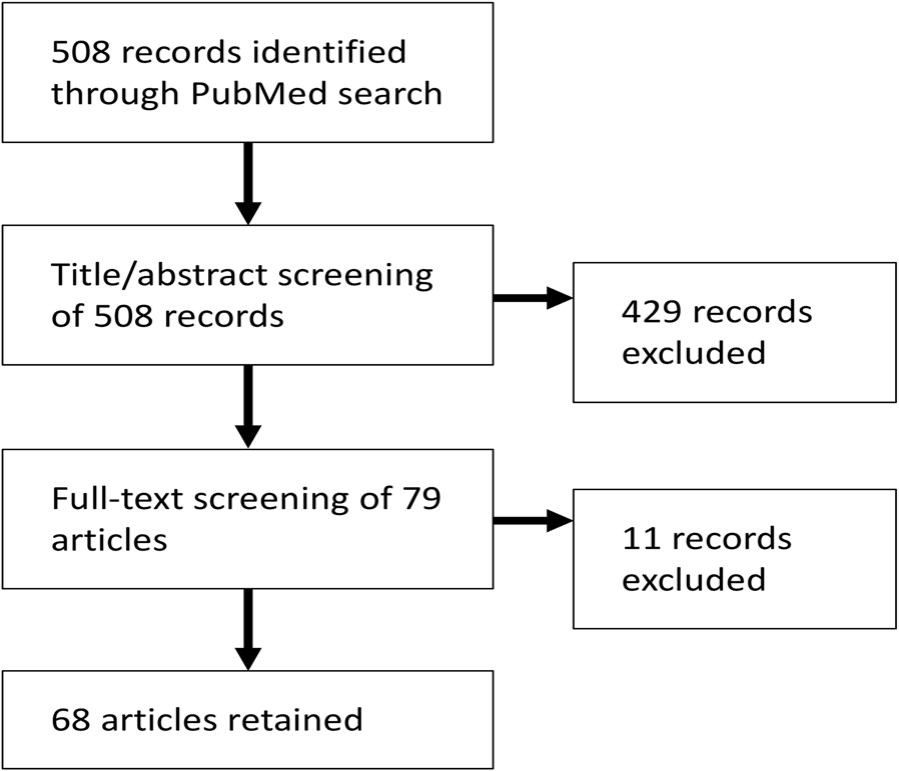
Literature review process to identify factors affecting health-related quality of life

We assessed the published research on predictors of HRQoL in PLHIV by utilising the aforementioned 2014 narrative review article on this topic and identifying original research articles published after the search period in the 2014 review. Forty-nine studies met the inclusion criteria for the 2014 review [25]. Additionally, our literature search identified 68 more recent studies reporting on factors associated with HRQoL in PLHIV (Fig. 1). Examples of relevant findings from the review article and the more recent studies are presented in Table 2. Numerous studies identified social support as a predictor of positive HRQoL outcomes. Physical and emotional health concerns, including symptoms, comorbidity, and depression, were often found to predict negative HRQoL outcomes. Other notable predictors of negative HRQoL outcomes included stigma, HIV disclosure concerns, and material insecurity (e.g., unemployment, financial problems, unmet needs for food and housing).

**Table 2:** Selected examples of study findings on factors associated with positive and negative health-related quality-of-life outcomes

Factors associated with health-related quality-of-life outcomes	Year	Source	Health-related quality-of-life instrument
*Factors associated with positive outcomes*			
Social support	2013	Bekele [52]	MOS-HIV
	2015	Dalmida [53]	SF-36
	2014	DeGroote [25]	N/A
	2019	den Daas [54]	SF-12
	2013	Emlet [55]	SF-8
	2016	George [56]	MOS-HIV
	2016	Niderost [57]	WHOQOL-HIV-Bref
	2013	Slater [58]	HAT-QoL
*Factors associated with negative outcomes*			
Comorbidity	2014	DeGroote [25]	N/A
	2013	Emlet [55]	SF-8
	2016	George [56]	MOS-HIV
	2016	Niderost [57]	WHOQOL-HIV-Bref
	2013	Slater [58]	HAT-QoL
Depression	2016	Ballester-Arnal [59]	MOS-HIV
	2017	Catalan [60]	WHOQOL-HIV Bref
	2015	Dalmida [53]	SF-36
	2014	DeGroote [25]	N/A
	2019	Olson [61]	FAHI
High symptom burden or presence of specific symptoms (e.g., body disfigurement, memory difficulties, sexual functioning)	2016	Ballester-Arnal [59]	MOS-HIV
	2017	Brandt [62]	WHOQOL-HIV-Bref
	2014	DeGroote [25]	N/A
	2019	den Daas [54]	SF-12
	2016	George [56]	MOS-HIV
	2019	Olson [61]	FAHI
Stigma	2018	Reinius [27]	Swed-Qual
	2013	Slater [58]	HAT-QoL
HIV disclosure concerns	2016	Fekete [63]	HAT-QoL
	2018	Logie [26]	SF-12
Material insecurity (e.g., unemployment, financial problems, unmet needs for food and housing)	2016	Ballester-Arnal [59]	MOS-HIV
	2014	DeGroote [25]	N/A
	2014	Douab [64]	SF-12
	2016	George [56]	MOS-HIV
	2018	Logie [26]	SF-12
	2016	Niderost [57]	WHOQOL-HIV-Bref
	2018	Sok [65]	MOS-HIV

FAHI: Functional Assessment of HIV Infection. HAT-QoL: HIV/AIDS-Targeted Quality of Life. MOS-HIV: Medical Outcomes Study HIV Health Survey. SF-8: 8-Item Short-Form Survey. SF-12: 12-Item Short-Form Survey. SF-36: 36-Item Short-Form Survey. Swed-Qual: Swedish Health-Related Quality of Life Survey. WHOQOL-HIV-Bref: World Health Organization Quality of Life–HIV BREF

### Qualitative study

Among service providers who participated in FGDs (N = 16), ten were men and six were women. The service providers included four HIV physicians, one psychiatrist, two psychologists, four nurses, one social worker, and four peers with diverse professional expertise. Among PLHIV who participated in FGDs (N = 15), eight were men, half of them men who have sex with men (MSM). Six were heterosexual cis women, and one was a transgender woman. Three PLHIV were immigrants, four were long-term survivors (diagnosed with HIV before 1996), and six were people who acquired HIV through injection drug use.

The analysis of focus group data identified several broad categories of issues that were thought to impact the health-related quality of life of PLHIV, with the most frequently mentioned issues falling into four categories: social problems; physical symptoms; psychological/emotional problems; and sexuality-related problems. Table 3 displays the categories and sub-categories identified, indicating the number of coded segments in each one, along with illustrative examples of segments.

**Table 3:** Issues impacting health-related quality of life of people living with HIV: analysis of focus group data

Category	Number of coded segments	Sub-category	Number of coded sub-category segments*	Illustrative example
Social problems	191	Anticipated stigma	150	I fear being rejected by my relatives. My mother has never rejected me and nor has my father, ever, but I do fear rejection by my relatives because they … they would never ever understand. [PLHIV Focus Group, Barcelona, woman] In everyday life, I’m very discreet [about having HIV] due to my fear of being discriminated against, or that people will be afraid that they will get infected or something weird like that. That they will have some strange reaction. [PLHIV Focus Group, Barcelona, man]
		Socioeconomic vulnerability	22	For these people, the biggest thing is their financial uncertainty. Some people live with very little money, extremely little, with pensions that get regularly reviewed. The infection becoming chronic, and the improvement of their physical health also have an impact on the reviewing of those pensions. In the past, if you had a pension, whatever it was, they never changed it, and that provided a certain future stability for that person. Now, that stability does not exist, and people feel vulnerable in that sense. [Experts Focus Group, Barcelona, physician] I haven’t worked in five years. The last jobs were temporary and ended unexpectedly. I’ve always thought that [the employers] knew something about my situation. [PLHIV Focus Group, Barcelona, man]
Physical symptoms	83	Sleep-related problems	28	There is another aspect that has always worried me in those patients and that is, for instance, from what I’ve been told, a certain emotional instability and lack of sleep, sleep disturbance, lack of rest.… They wake up, especially [when newly diagnosed with HIV], … they have told me that they wake up often; they are concerned; they don’t rest well. [Expert Focus Group, Madrid, physician] Either I have trouble sleeping or I wake up in the middle of the night and go two hours without sleeping. It depends on the week more or less, on whether I have a lot of problems at work which keep me up at night because I can’t get them out of my head. [PLHIV Focus Group, Barcelona, man]
		Fatigue	17	My main problem is tiredness. I have very severe chronic fatigue…. I had larynx cancer in 2012, and after radiotherapy, my energy levels never went back up.… My current struggle is to have a good quality of life and to rest. The company [where I work] has moved its headquarters to Sant Joan Despí, so I need to catch a train at six in the morning and I get back home at four in the afternoon. Then I lie down in bed like a mummy after lunch to rest, because I can’t … I can’t manage this pace. [PLHIV Focus Group, Barcelona, woman] I have felt physically tired for a long time…. I have felt that it is difficult for me to start the day, that I’d rather not wake up, that moment between when I wake up and the activity begins, I wish I didn’t exist…. I attribute [the tiredness] to the fact that I am recovering from depression, but … it’s a physical thing. [PLHIV Focus Group, Barcelona, man]
		Physical pain	14	Persistent chronic pain. I think that we should assess and thoroughly detail what type of pain, whether articular or muscular or.… To talk about it in terms of the degree of pain perceived by every single person. However, general pain is something that is often brought up at doctor’s appointments. [Expert Focus Group, Madrid, physician] For a couple of years my bones have hurt a lot. I’ve mentioned it to the doctor and it could be the effect of the medication, but he doesn’t really confirm this. [PLHIV Focus Group, Barcelona, man]
		Body fat changes	10	There is still a group of people who are survivors of a different era.… They may have body changes and, I am not sure if this is subjective, [but] women feel that it affects them more than men.… That is the feeling I have from the consultations. Another prevalent problem in some cases, more and more prevalent, is the uncontrolled weight gain, you know? Sometimes, for good reason, there are many totally unusual diets … high caloric intake, carbonated drinks… [Expert Focus Group, Barcelona, physician] I began to notice that I was losing weight in my legs because I’ve always been athletic and had good muscles…. But I began to lose fat there, I began to notice my veins.… I said “Oh my God! What’s happening to me?” I went to the doctor, but the doctor did not resolve the matter. And for me, well, that affected me tremendously.” [PLHIV Focus Group, Barcelona, transgender woman]
Psychological problems	67	General emotional distress	43	One patient said to me: “It’s not only physical. It’s not just that my feet hurt because I have neuropathy. It’s that I haven’t developed myself. My soul hurts. I don’t have a job like everyone else, nor a pension. I feel old…. I don’t really have anything.” [Expert Focus Group, Barcelona, physician] I think there is emotional discomfort linked to low self-esteem. For the person living with HIV, there are a thousand reasons, sometimes multifactorial but then linked to the sense of belonging or loss of social skills, with greater isolation, with drug use, unhealthy behaviours.... All of this is linked either by the path that the person had at the start, which is later somehow provoked by the diagnosis of HIV, or just by the diagnosis of HIV itself. [Experts Focus Group, Madrid, peer educator]
		Anxiety/depression	24	You can’t sleep, sometimes you panic, you know? That feeling that when I go to bed: *Ahh!* [Inhaling.] I feel like I suffocate. And then it’s like if you wake up: *Ahh!* [Inhaling.] And it is, it is horrible. It’s horrible. [PLHIV Focus Group, Barcelona, man] For me, one of the most important problems that negatively affects quality of life is mental health, with anxiety and depression being the most prevalent problems. They can also be multifactorial. [Expert Focus Group, Barcelona, physician]
		Fear of the future	14	An issue, mostly among people who are growing old, is concern about the future. Because if, on top of that, you have financial issues and social issues, the concern about the future and the uncertainty about what is going to happen to you … impacts a lot on the quality of life of people with HIV. [Expert Focus Group, Barcelona, psychologist] I have a family history of dementia, also a case of Alzheimer’s [in my family], and at the age of 64, well, when I forget something.… I think that tomorrow I’ll be like my mother, who is 91 and doesn’t remember anything. That is the great fear that I have. It’s maybe what worries me the most. [PLHIV Focus Group, Barcelona, man] Some of the people I was close to, family or other relationships, slowly disappeared.… You see yourself alone and think, “How am I going to deal with this?” [PLHIV Focus Group, Barcelona, man]
Sexuality-related problems	38	Lack of libido	9	Sexual health issues. Not only sexually transmitted infections … but also issues related to the loss of libido. Especially for women. It’s a very important issue that affects their quality of life. [Expert Focus Group, Barcelona, nurse] The problems of lack of libido, lack of sexual desire – especially in women. Especially in women who have been living with HIV for many years. It is quite common when you talk with women that they tell you about this problem. [Expert Focus Group, Barcelona, NGO professional]
		Sexuality and HIV-related stigma	5	For years now, I’ve given up on sexual issues, only … focusing on masturbation. Because I also had that feeling of guilt, right? I said, “If I like someone, they want me … I also want them, but I feel bad, right? How am I supposed to tell them?” [PLHIV Focus Group, Barcelona, man] Despite the available information, people anticipate the fear of, “How am I going to establish a sexual relationship when I have HIV? Do I say it or do I not say it?” It is often the main issue when dealing with sexuality. [Expert Focus Group, Barcelona, nurse]

*Segments could be coded in more than one subcategory depending on the content. Thus, the number of segments coded in each category does not necessarily correspond to the sum of the segments coded in that subcategory. Not all subcategories are reported in this table

#### Social problems

Within the category of social problems, stigma was by far the issue raised the most frequently (Table 3). In the FGD prioritisation exercise, stigma/discrimination was ranked as the most burdensome issue by both service providers and PLHIV.

While a number of different aspects of stigma and discrimination were discussed, many of these related to the concept of anticipated stigma, i.e., the belief that one’s HIV-positive status would elicit negative responses in other people. Anticipated stigma was reported to have important implications for many PLHIV in regard to social and intimate relationships. Among other concerns, a number of PLHIV focus group participants emphasised the effects of anticipated stigma on their romantic and sexual lives. Self-stigma often was noted to be part of the dynamic when these issues were discussed. Service providers called attention to the interconnected nature of public stigma, self-stigma, and a broad range of other challenges that PLHIV experience.

Another social problem, socioeconomic vulnerability, was identified as a high priority by service providers, but not by PLHIV. Service providers noted both the immediate financial challenges facing PLHIV and also the concerns that some PLHIV expressed about not being able to accumulate the financial resources that they will need in later life. Service providers reported having the perception that some PLHIV patients may be experiencing financial difficulties although this issue is seldom discussed explicitly in clinic visits. One service provider also called into question the stereotype that PLHIV who belong to the MSM community are financially secure.

#### Physical symptoms

Within the physical symptoms category, the issues named most frequently were sleep-related problems and fatigue (Table 3). PLHIV spoke about these issues being a prominent part of their lives. It was also observed that sleep-related problems were not being resolved satisfactorily.

Physical pain also was frequently mentioned by both PLHIV and service providers. Physical pain was identified as a cause of sleep problems. One PLHIV focus group participant, for example, reported being awakened several times per night by shoulder pain.

PLHIV and service providers also noted concerns about body fat changes such as lipoatrophy and weight gain. Multiple PLHIV focus group participants reported that body fat changes had been emotionally distressing. One service provider expressed the view that improvements in ART have meant that only a very small proportion of PLHIV continue to suffer from body fat changes. Another service provider, however, called for continuing attention to this issue.

#### Psychological problems

In the category of psychological problems, FGD participants most commonly spoke of emotional distress in general terms rather than naming specific disorders. When people commented about specific disorders, the ones most frequently mentioned were depression and anxiety. Fear of the future was identified as an issue negatively affecting the emotional health of some PLHIV. In the prioritisation exercise, both service providers and PLHIV ranked psychological well-being as the second-most important issue following stigma. The burden of mental health challenges experienced by PLHIV was emphasised in all focus group discussions. One service provider called for emotional well-being to be viewed as more than the absence of conditions such as depression and anxiety. Service providers noted that the aging experience may bring on further mental health challenges.

#### Sexuality-related problems

Focus group participants mentioned sexuality-related problems considerably fewer times than they mentioned other issues such as stigma and physical symptoms (Table 3), but sexuality-related problems were ranked as the third-most burdensome issue in the prioritisation exercise. More service providers than PLHIV identified this as a high-priority issue. Service providers also talked about sexuality-related problems more than PLHIV did in the focus group discussions. Comments from service providers as well as PLHIV touched on a wide range of concerns, including sexually transmitted infections, hormonal problems, lack of libido, and general sexual dissatisfaction. When PLHIV spoke about sexuality, their concerns often encompassed the issue of HIV-related stigma.

## Discussion

This article describes the formative research phase in the development of the CST-HIV, a novel PROM that is intended to help healthcare providers identify burdensome health-related problems experienced by PLHIV in routine clinical care in Spain. The literature review and focus group findings identified a range of concerns that subsequently informed decision-making about the content of the CST-HIV. These issues span the domains of physical, emotional and social well-being, reflecting the complexity of living with HIV as a long-term chronic condition. Findings contribute to documenting the content validity of the CST-HIV, thus addressing one of the key criteria for instrument development [66, 67].

Quantitative research findings corroborate the importance of many of the issues that focus group participants identified as factors negatively affecting the HRQoL of PLHIV. For example, studies have indicated that half or more of PLHIV experienced pain in recent months [68], and pain is undertreated in PLHIV [69, 70]. PLHIV are five times more likely than HIV-negative people to suffer from insomnia, which often remains undiagnosed [71]. A systematic review of depression in PLHIV reported an estimated point prevalence of 33%, which appears to be much higher than the prevalence of depression in the general population [72, 73]. Anxiety and sexuality-related problems are also more prevalent in PLHIV than in the general population [72, 74]. Half of PLHIV are thought to experience sexuality-related problems, and these problems are often not recognised by physicians [74, 75].

A striking finding of our qualitative research was the strong emphasis that focus group participants placed on the role of stigma and discrimination in reducing health-related quality-of-life. Not only was stigma/discrimination the highest-ranked issue when PLHIV and service providers were asked to identify the issues that they believed to be most burdensome, but it was also addressed far more often than any other issue in the focus group discussions. HIV-related stigma takes multiple forms, including anticipated stigma, internalised stigma and enacted stigma (i.e., manifestations of stereotyping, prejudice or discrimination) [76]. Interestingly, our focus group discussions showed considerably more concern about anticipated stigma than other forms of stigma.

HIV-related stigma in general is reported to be common and distressing among PLHIV worldwide [72, 77, 78]. HIV-related stigma is associated with multiple health outcomes of concern, including lower ART adherence, lower usage of health and social services, poorer physical health, and worse HRQoL outcomes [79, 80]. The existence of evidence-based interventions that can mitigate some of the negative effects of HIV-related stigma, as well as evidence-based interventions that can reduce HIV-related stigma itself [81], provides a strong rationale for healthcare providers to seek to identify PLHIV who are experiencing anticipated stigma and other forms of stigma. These patients may benefit from screening for associated problems and from referral to a range of health and psychosocial services.

As focus group participants themselves suggested, many of the issues that undermine HRQoL in PLHIV may be interrelated. For example, pain and sleep problems are often found to co-occur [82], and both of these issues are associated with depression in PLHIV [68, 83]. Associations also have been observed between HIV-related stigma and sexuality-related problems [84], and between both of these issues and depression [74, 79]. While it is difficult to determine the cause-effect relationship in many of these associations, we speculate that successfully addressing some of these issues in clinical care may reduce the burden of other related issues. Thus, a PROM such as the CST/HIV may assist healthcare providers in identifying multiple pathways through which various factors affecting the HRQoL of PLHIV can be influenced.

The formative research reported in this article directly guided the definition of constructs and development of items for the CST-HIV, which was subsequently piloted and found to have adequate psychometric properties. The eight domains of the 24-item instrument closely reflect literature review and FGD findings (Box 1) [40]. Commonalities and differences can be observed when the CST-HIV is compared to two other short, broadly focused PROMs developed to support the clinical care of PLHIV: the Positive Outcomes PROM and the Short-Form HIV Disability Questionnaire (SF-HDQ) [39, 85]. The framing of the SF-HDQ in terms of disability may seem to imply a narrower purpose for this instrument compared to the two others, but in fact the developers of the SF-HDQ based their work on a highly comprehensive definition of HIV-associated disability as “a combination of physical, cognitive, mental and emotional symptoms and impairments; difficulties carrying out day-to-day activities; challenges to social inclusion; and uncertainty about future health” [86]. This focus overlaps considerably with the focus of the CST-HIV and the Positive Outcomes PROM.

**Box 1:** Domains of the HIV Clinic Screening Tool [40]

• Anticipated stigma	
• Emotional distress	
• Sexuality	
• Social support	
• Material deprivation	
• Sleep and fatigue	
• Cognitive problems	
• Physical symptoms	

The CST-HIV, Positive Outcomes PROM and SF-HDQ all address physical, emotional and social well-being, although the ways in which they do so reflect varying degrees of concern with some specific health-related issues. The CST-HIV is the only one of the three instruments with domains for sleep/fatigue, material deprivation, sexuality, and anticipated stigma [40]. The other instruments address facets of these issues: the SF-HDQ, for example, has an item about the respondent’s ability to “maintain safe and stable housing,” and the Positive Outcomes PROM has an item about HIV disclosure [39, 85]. The Positive Outcomes PROM is the only instrument with items about information needs (1 item), drug/alcohol use (1 item), immigration concerns (1 item) or contraception concerns (1 item) [85].

Multiple factors may account for these and other differences, such as methodological differences in the instrument development processes and researcher biases favoring the prioritisation of some issues over others. An additional possibility is that HIV-related care priorities may vary across geographical settings in accordance with social, cultural, epidemiological or health system differences. The qualitative data that guided the selection of domains for the Positive Outcomes PROM were gathered from interview participants in the United Kingdom and Ireland, while the qualitative data that informed the theoretical framework for the SF-HDQ were gathered from focus group participants in Canada [34, 87].

To give one example of how priorities may vary, Spanish FGD participants occasionally expressed concerns about problematic alcohol use but did not identify this as a highly burdensome issue, as reflected in our reporting of the FGD findings and prioritisation exercise. Researchers have called attention to wide variation in cultural norms regarding alcohol consumption, even among Western European countries [88, 89]. Furthermore, it has been proposed that cultural norms also influence perceptions about the incidence and prevalence of alcohol dependence and alcohol use disorders, even when standardised instruments are being used to assess these issues [89]. In this context, it is notable that prevalence of alcohol dependence is estimated to be higher in both England (6.9%) and Ireland (4.2%) than in Spain (0.7%) [90], and that the Positive Outcomes PROM unlike the CST-HIV includes an item about alcohol use [85].

The COVID-19 pandemic, which emerged midway through the CST instrument development process, has become a notable part of the context of long-term HIV care and underscores the importance of focusing on issues beyond viral suppression of HIV. A longitudinal study using the HIV Disability Questionnaire found that PLHIV in Canada reported more severe disability experiences following the onset of the COVID-19 pandemic, including in the physical, mental-emotional and social domains [91]. Given the pandemic’s long-term impact on many individuals’ financial resources, social support systems and access to medical care, it may be the case that some high-priority health-related issues reported by our focus group participants in 2019 have subsequently become more burdensome for PLHIV in Spain and elsewhere. At the same time, the depletion of health system resources during the first two years of the COVID-19 pandemic has intensified the need for clinical tools that enable healthcare providers to efficiently identify and focus on patients’ most pressing concerns. There is now an even greater imperative to explore how PROMs such as the CST-HIV may contribute to improving clinical care and achieving better health and HRQoL outcomes.

This study has a number of limitations. The literature review that constituted the first stage of the instrument development process was not systematic, thus possibly reducing the number of relevant studies identified. Participants in the service provider FGDs were recruited from among the professional networks of the researchers who led this study, and unknown biases may have influenced their selection. Participants in the PLHIV FGDs were recruited from among clients at a Barcelona-based NGO and patients at a large Barcelona university hospital. Although purposive sampling ensured sociodemographic diversity in both of the PLHIV FGDs, the PLHIV who participated in this study might not be representative of PLHIV in other settings in Spain in regard to the health-related issues that they identified as most burdensome. PLHIV might have felt reluctant to discuss some sensitive personal matters in a group setting, and thus the PLHIV focus group findings might not accurately convey the extent of the burden imposed by issues such as problematic drug and alcohol use, financial hardship and sexuality-related problems. Given the heterogeneity of the Spanish PLHIV population [92], conducting a larger number of focus groups may have yielded more information, particularly in relation to intersectional issues faced by many PLHIV.

## Conclusion

Various types of health-related issues have the potential to undermine the HRQoL of PLHIV, including physical symptoms, psychological problems, sexuality-related problems, stigma, and socioeconomic vulnerability. Rigorous qualitative research should guide the development of PROMs that can be used by healthcare providers to effectively identify the most burdensome health-related issues experienced by their patients. Future research on how PROMs might contribute to HIV care should explore whether there are notable variations across countries regarding which health-related issues are perceived to be the most burdensome.

## Data Availability

All relevant data are within the paper. Anonymized literal transcripts of interviews may be requested from the authors.

## Annex 1: Search strings used in literature search

### Symptom burden in people living with HIV

(((HIV OR AIDS) AND "symptom burden")) AND ("2010"[Date - Publication]: "3000"[Date - Publication])

(((HIV OR AIDS) AND symptom list)) AND ("2000"[Date - Publication]: "3000"[Date - Publication])

((symptom cluster*[Title]) AND (((HIV[Mesh] OR "Acquired Immunodeficiency Syndrome"[Mesh] OR HIV OR AIDS)))) AND ("2010/01/01"[Date - Publication]: "3000"[Date - Publication]).

### Health-related quality of life in people living with HIV

((“quality of life”[Title]) OR (quality-of-life[Title]) OR (QOL[Title]) OR (“health-related quality of life”[Title]) OR (“health-related quality-of-life”[Title]) OR (HRQOL[Title]) OR (“life quality”[Title])) AND ((HIV[Title]) OR (“human immunodeficiency virus”[Title]) OR (“acquired immunedeficiency syndrome”[Title]) OR (“acquired immune deficiency syndrome”[Title]) OR (AIDS[Title]) OR (“people living with HIV” [title]) OR (PLWHIV[Title]) OR (PLWH[Title]) OR (PLHIV[Title]) OR (HIV[Mesh]) OR (“Acquired Immunodeficiency Syndrome”[Mesh])) AND (“2013"[Date - Publication]: “3000”[Date - Publication]).

## Annex 2: Script for focus group discussions

### Introduction

1. [Introductions]
2. [Thank participants for coming]
3. We are conducting this focus group as part of a research project to develop a short clinic screening tool for PLHIV. This project is being carried out by ISGlobal in collaboration with SEISIDA and is part of a wider research project aimed at improving the quality of life of people with HIV.
4. [Confidentiality – tell participants appropriate information]
5. [Tape recording – tell participants appropriate information]
6. [Ask participants to read and sign informed consent forms]
7. [Tell participants other practical details: (a) please turn off mobile phones; (b) the discussion will last for approximately 90 min without a break]
8. Does anybody have any questions before we begin?

### Focus group questions

*Before asking the first question, explain more about the clinic screening tool:*

The purpose of the clinic screening tool is to help healthcare providers identify the most burdensome health-related problems that PLHIV in Spain are experiencing. By “burdensome” we mean the health-related problems that have the greatest negative impact on their quality of life.

We are defining health-related problems broadly. These problems may include symptoms, comorbidities and medication side-effects. They may relate to physical and mental health. Our definition of health-related problems also includes psychosocial and interpersonal issues that affect the health of PLHIV, for example, housing insecurity, social isolation and stigma.

In this focus group discussion, we want you to tell us your thoughts about the most burdensome health-related problems experienced by PLHIV in Spain. This will help us to identify items that should be included in the clinic screening tool. We will also be asking you if you know about any effective interventions for addressing these problems.

**FGQ-1 Experts.** **In your opinion, what health-related problems have the greatest negative effect on the quality of life of people with HIV?**

**FGQ-1 PLHIV.** **What health-related problems have the greatest negative effect on your quality of life?**

**Prompts to facilitators:**

- *Use a large flip chart to list responses.*
- *Listen for responses in all three of our domains of interest – symptoms, comorbidities and psychosocial/interpersonal. If the conversation is focusing on one domain, for example symptoms, prompt the group to consider another domain by asking:*
  -   **What about comorbidities?**
  -  **What about psychosocial and interpersonal issues?**

- Be prepared to prompt the group about any major issues that participants may be forgetting. For example:
  -  **Nobody has talked about self-stigma. Is this a burdensome health-related problem for PLHIV in Spain?**

*The following table may help with prompts:*

**Table:**

Symptoms	Comorbidities	Psychosocial/interpersonal
Sleep problems	Depression	Self-stigma
Chronic pain	Anxiety	Discrimination by healthcare providers
Fatigue	Alcohol/drug dependence	Unstable housing
Sexual dysfunction		Financial insecurity
Memory or other cognitive problems		Medication adherence concerns
		Social isolation
		Unmet need for pregnancy planning

**After they have listed or named problems, focus group participants should be asked to define the dimensions of the problems.** *Example: You mentioned that sleep problems are a common and relevant problem. Could you explain in some detail how this problem manifests?*

**FGQ-2_Experts.** **Among the health-related problems that you have identified, which ones are most important to include in a short clinic screening tool?**

- *Ask focus group participants to indicate priorities on flip-chart lists using green stickers*

**FGQ-2_PLHIV.** **Of all the problems you have mentioned, what do you think are the most important considering not only you but people with HIV in general?**

- *Ask focus group participants to indicate priorities on flip-chart lists using green stickers*

**FGQ-3_Experts.** **Are you generally able to identify any of these problems in your patients? If yes, how?**

- *The focus should be on the priority problems identified in FGQ-2, not on all problems that have been named*.
- *Ask focus group participants if they are using a PROM to identify priority problems – if yes, which one?*

**FGQ-3_PLHIV.** **Do you discuss these problems with your doctor or other healthcare provider?**

***If yes:***

- **How does the issue come up in the consultation – does the doctor make the diagnosis of that problem in the consultation? Or are you the one bringing up the problem?**
- **Does your healthcare provider do anything to help you to solve or reduce this problem?**

***If no:***

- **Why don't you talk about it?**

**FGQ-4_Experts.** **What interventions do you make to respond to these problems?**

**FGQ-4_PLHIV.** **What do you think your doctors or other health professionals could do to help you solve or reduce those problems you mentioned?**

**FGQ-5_Both.** *This will be the last question – its purpose is to bring up any important issues that have not been addressed.* **We have about XX minutes remaining. Does anyone want to add anything to this discussion about burdensome health-related problems in PLHIV?**

**END (5 minutes)**

Thank you very much for your time. You are welcome to follow up with us by e-mail if there is more information that you want to add later.

## Abbreviations

ART: Antiretroviral therapy
CST-HIV: HIV clinic screening tool
FGDs: Focus group discussions
HRQoL: Health-related quality of life
MSM: Men who have sex with men
NGO: Nongovernmental organisation
PLHIV: People living with HIV
PROMs: Patient-reported outcome measures
